# Histopathological comparison of colorectal neoplasia or polyps development between young adults and older adults: Our experience of 735 consecutive cases and 1269 polyps

**DOI:** 10.12669/pjms.40.6.8475

**Published:** 2024-07

**Authors:** Ali Koyuncuer

**Affiliations:** 1 Dr. Ali Koyuncuer, MD, Associate Professor, Pathologist, Department of Pathology, Umraniye Training and Research Hospital, University of Health Sciences, Istanbul, Turkey

**Keywords:** Adenoma, Conventional, Dysplasia, Intramucosal Carcinoma, Polyps, Serrated

## Abstract

**Objective:**

In recent decades, there has been an increase in early-onset colorectal cancer, the need to screen individuals younger than 50 years of age, and the presence of histopathological differences remains unclear. The objective of this study was to explore the occurrence of polyps in both young adults and older individuals and to examine their potential correlation with colorectal cancer.

**Methods:**

In this retrospective study conducted between July 1, 2018, and October 5, 2022, in the Pathology Laboratory, we designed a study based on the histopathological features of colorectal polyps evaluated by an experienced gastrointestinal pathologist based on the WHO 2019 classification.

**Results:**

We evaluated 735 consecutive patients who underwent colonoscopic polypectomy between July 2018 and October 2022. The prevalence of cases under the age of 50 was 13.9%, and adults over the age of 50 was 86.1%. A total of 1269 polyps were detected, 1215 (95.7%) were epithelial polyps and 145 (11.9%) were epithelial polyps under the age of 50. One hundred four conventional adenomas and four intramucosal carcinomas were detected in cases younger than 50 years. The patients in the low-risk adenoma group was 57%, and the rate of patients in the high-risk adenoma group was 14.9%. Overall, polyps were most common in the sigmoid colon and there was a statistically significant difference between detecting tubular adenomas in the sigmoid colon (P=0.04).

**Conclusions:**

Our current results confirm the detection of sporadic colorectal adenomas and advanced neoplasia in young adults.It is important to establish professional community guidelines for surveillance colonoscopy in these age groups.

## INTRODUCTION

Colorectal cancer (CRC) is the third most common cancer in the United States, with approximately 135,000 new cases reported each year. It is the second leading cancer death and causes about 50,000 deaths annually.^1^,^2^ Colonoscopic screening and surveillance studies have increased with the demonstration that the majority of CRCs develop via the adenoma-carcinoma pathway, but it continues to have an important place in cancer deaths in the United States.^3^,^4^ The American College of Gastroenterology Guidelines recommends performing a colonoscopy every 10 years starting at age 50 as the preferred CRC prevention test. The American Cancer Society (ACS) recommends that adults aged 45 years and older with an average risk of CRC should be tested regularly with stool-based tests or visual examination, and if a positive test is detected, they should undergo colonoscopic follow-up. ACS recognizes that screening from age 45 is a qualified recommendation.^2^,^5^ Although it is routine in young adults, the number of colonoscopies is increasing, albeit on a symptom-based basis. A recent study observed that colonoscopy rates increased gradually between the ages of 45-54, but at the same time, cancer rates in the 40-44 and 45-49 ranges increased.^6^ These guidelines are limited if an adenoma is detected in a young adult patient. It has been reported that there may be different mechanisms associated with the development of colorectal cancer in patients under the age of 50.^7^ Therefore, the detection of conventional adenoma in young adults may mean an increased risk for the development of neoplasia in the future. Patients under 50 years of age are defined as young adults with early-onset lesions. Our study aimed to reclassify early-onset polyps of young adults and elderly patients and their relationship to CRC using WHO-2019.

## METHODS

In this retrospective study, we examined histopathologically the specimens of consecutive patients who underwent total colonoscopy and polypectomy for the first time sporadically between July 1, 2018, and October 5, 2022, in the Pathology Laboratory, which tertiary health care institutions with 836. All polyps were reclassified according to the WHO-2019 classification of digestive system tumours according to histological types and demographic characteristics.

Ethical Approval: It was obtained from the Health Research Ethics Committee (under protocol number: B.10.1.TKH.4.34.H. GP.0.01/ 212 and 203).

The patients were divided into main groups according to their ages 18-29, 30-39, 40-49, and ≥50 years. Advanced colorectal neoplasia (ACRN) was defined as detected cancer or advanced adenoma. All adenomas ≥10 mm, tubulovillous or villous architecture, and/or high-grade dysplasia (HGD) or intramucosal adenocarcinoma were defined as advanced adenoma. All cases were categorized histopathologically as low-risk, intermediate and high-risk adenomas. A low-risk adenoma or group was defined as patients with 1-2 tubular adenomas <10 mm in size, intermediate risk (3-4 tubular adenomas <10 mm), high-risk adenoma or group was defined as adenomas ≥10 mm, and patients with ≥5 or more adenomas, advanced adenoma, and high-grade dysplasia adenomas with villous/tubulovillous histology. Polyps were classified according to their size as diminutive, small, medium, and large; <5 mm, 6-9 mm, ≥10-19 mm, and ≥20 mm respectively.

### Statistical analysis

Colorectal polyps were classified histopathologically and divided into young adults and older adults. Standard deviation (SD) was used for continuous variables, and numbers or percentages were used for categorical variables. Clinical features were evaluated with the Pearson chi-square test for continuous variables and categorical variables, the student-t test for normal distribution, the nonparametric Kruskal-Wallis test (≥3 groups), and Mann–the Whitney test (two groups) for abnormal distribution. SPSS version 22 was used for statistical analysis (SPSS Statistics for Windows; IBM, Armonk, New York, USA). A P-value ≤ 0.05 was considered statistically significant.

## RESULTS

A total of 1269 polyps from 735 consecutive individuals undergoing colonoscopy were examined histopathologically. The mean age of all subjects included in the study was 61.2 (11.1) years. Of all cases, 50 or older were 633 (86.1%) and the mean age was 64.2 years, younger than 50 patients were 102 (13.9%), mean age was 42.2 years. Of the cases, 441 (60%) were males and 294 (40%) were females (Table-I). There were 513 cases with polyps or lesions in the right colon, and 756 in the left colon and rectum (Table-II). Of all polyps, 1215 (95.7%) epithelial polyps, 37 (2.9%) inflammatory polyps, and 17 (1.3%) mesenchymal polyps were detected. Mesenchymal polyps were not observed under 50 years of age, and inflammatory polyps were predominantly observed over 50 years of age. We did not find a statistically significant relationship between the histomorphologic features of epithelial polyps and age groups (P=.846), but we observed statistical significance between hyperplastic polyps and the 30-39 years age group (P=.008). The incidence of tubular adenomas derived from conventional adenomas in the proximal colon compared to the distal colon was statistically significant (P=.000).

**Table-I:** Histopathological findings of polyps according to the basic characteristics and age groups of the patients

Characteristic	18-29 (n=3)	30-39 (n=28)	40-49 (n=71)	≥50 (n=633)
Age, years	27.67±1.53	35.75±2.91	45.39±2.47	64.26±8.39
Gender, male	27.5 (2)	35.62 (13)	45.38 (45)	63.97 (381)
Total, n=1269 polyps	0.23 (n=3)	2.91 (n=37)	8.66 (n=110)	88.18 (1119)
Colorectal sessile serrated lesions				
Hyperplastic polyp	0.00 (0)	1.10 (n=14)	1.49 (n=19)	14.49 (n=184)
SSL-D	0.00 (0)	0.00 (0)	0.31 (n=4)	1.73 (n=22)
TSA	0.00 (0)	0.00 (0)	0.00 (0)	0.63 (n=8)
USA	0.00 (0)	0.00 (0)	0.00 (0)	0.07 (n=1)
TSA+Adca	0.00 (0)	0.00 (0)	0.00 (0)	
Conventional colorectal adenomas				60.52 (n=768)
Tubular adenoma, low grade	0.23 (3)	1.65 (n=21)	5.83 (n=74)	4.57 (n=58)
Tubulovillous adenoma, low grade	0.00 (0)	0.07 (n=1)	0.39 (n=5)	0.55 (n=7)
Villous adenoma, low grade	0.00 (0)	0.00 (0)	0.00 (0)	1.41 (n=18)
Advanced adenoma (intramucosal ca)	0.00 (0)	0.00 (0)	0.31 (n=4)	0.23 (n=3)
Invasive adenocarcinoma	0.00 (0)	0.00 (0)	0.00 (0)	
Inflammatory Polyps	0.00 (0)	0.07 (n=1)	(n=4)	2.52 (n=32)
Mesenchymal Polyps	0.00 (0)	0.00 (0)	0.00 (0)	1.33 (n=17)

SSL-D: Sessile serrated lesion with dysplasia, TSA: Traditional serrated adenoma, USA: Serrated adenoma, unclassified, TSA+Adca: Traditional serrated adenoma synchronous adenocarcinoma.

**Table-II:** The anatomic distribution of colorectal polyps and lesions.

	n	%
Proximal colon	513	40.42
Distal colon	756	59.58
Total	1269	100
Cecum	62	4.9
Ileocecal valve	6	0.5
Ascending colon	179	14.1
Hepatic flexure	61	4.8
Transverse colon	205	16.2
Splenic flexure	34	2.7
Descending colon	205	16.2
Sigmoid colon	277	21.8
Rectosigmoid region	22	1.7
Rectum	218	17.2

Total	1269	100

**Fig.1:**
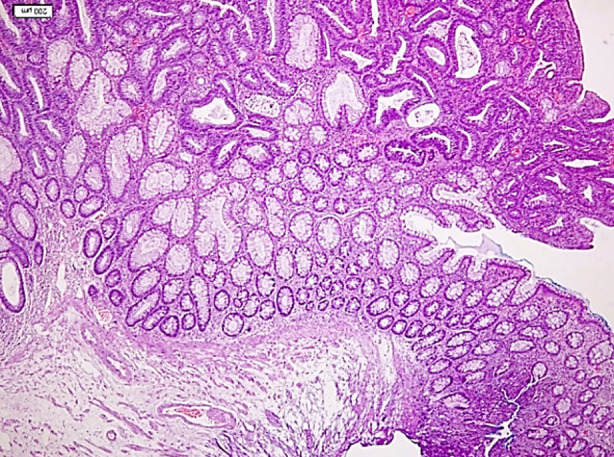
Conventional tubular adenoma. Compare with polyp and non-dysplastic colonic mucosa (bottom right of image) composed of tubules and adenomatous epithelium showing predominantly low-grade dysplasia (Hematoxylin-eosin stain, X10 objective).

We observed statistically significant incidence of hyperplastic polyps in the sigmoid colon and rectum compared to other colon segments (P=.005 and P=.005). The mean number of polyps was 1.72 (1.3), the mean number was 2.9 (1.5) in those with synchronous neoplastic polyps, and the mean size was 5.6 (5.8) mm. Seventy percent of all polyps were diminutive polyps (≤ 5 mm), 15% were small polyps (6-9 mm), 11.4% were medium-sized polyps (10-19 mm), and 2.9% were large polyps (≥20 mm). However, we found a statistically significant correlation between tubulovillous adenomas larger than 8 mm, particularly 13 mm and 15 mm and larger (P=0.026 vs. P=0.044 vs. P=0.000). We observed a statistically significant correlation between them with 13 mm for TSAs, 17 mm for SSLs and 25 mm for advanced adenomas (particularly those with intramucosal carcinoma) (P=0.016, P=0.024, P=0.031). The prevalence of 1–2 tubular adenomas, polyps <10 mm in size, and low-risk adenomas/lesions with SSL low-grade dysplasia was 57% (n=421 cases). The rate of patients with high-risk polyps ≥10 mm, villous, tubulovillous (Fig.1), and TSA histology was 5.7% (n=42 cases), and the prevalence rate of adenocarcinoma within the polyps and advanced adenoma was 2% (n=25) all epithelial polyps. Synchronized invasive adenocarcinoma was also present in another segment of the colon-rectum in 1% (n=7) of all cases. 28% of the patients (n=208) between 50 and 59 years old, with more than half of the patients being about 58% (n=427) older than (≥) 60 years. 64% (n=41) of tubulovillous adenomas, 85.7% (n=6) of villous adenomas, 63.6% (n=14) of advanced adenomas, 66.6% (n=2) of invasive carcinomas older than 60 years.

## DISCUSSION

In our research, we aimed to investigate the occurrence of colorectal polyps and their relationship with cancer in patients both above and below 50 years of age. Additionally, we explored their correlation with demographic factors, polyp size distribution, and histopathological characteristics. It is noteworthy that there is a scarcity of histopathological studies specifically addressing this topic in the existing literature; most available studies tend to be either epidemiological or public health-focused. Our research uncovered a notable rise in the prevalence of colorectal adenomas among individuals under the age of 50. Particularly significant was the increase observed around the age of 42, marking it as a pivotal cumulative age. These findings underscore the rationale for initiating colorectal screening at the age of 45. Today, the standard practice for colonoscopy is the resection of all detected polyps and their histopathological evaluation. Determining future colonoscopic surveillance is based on the number of adenomas, the size of the polyps, advanced histomorphological features in the polyps, and the presence of invasive carcinoma. Currently available guidelines for colorectal carcinomas recommend starting screening at age 50 or older. On the other hand, colorectal carcinomas detected in young adults differ in that they are more aggressive and in more advanced stages compared to older adults. The screening or treatment of individuals under the age of 40 with a personal history of colorectal neoplasia or polyps is clearly stated in the national guidelines. However, there is less data on how to manage it in young adults with no family history and incidentally detected adenoma.^8^ In the study of Momenti et al., which included 1623 people, it was determined that approximately 5.6% (n=92) of the cases were under the age of 50, and 94.4% of them were over the age of 50. The mean age was determined as 45 vs 67. Both age groups consisted of predominantly male (>60%) patients.^9^ We found that the rate of cases older than 50 years was 86.1%, that of those under 50 years old was 13.9%, and the median age is 64 vs. 42 years. The early detection rate in these results can be explained by the easy access to health care services and doctors in our hospital. The findings on male dominance in the study are supported by the literature. A comprehensive histopathological classification study on the prevalence of colorectal adenomas and carcinomas has not yet been conducted in our country. Interestingly, 13.9% of cases with at least one polyp were found to be under the age of 50, which aligns with the findings from The National Polyp Study. This result underscores the importance of conducting cohort studies to address large populations in colorectal cancer screening. On the other hand, Kwak et al. reported that the prevalence of adenoma was found to be higher in the 30-39 age range compared to younger age groups (about 12.6%), 13.1% in men, and 7.7% in women in this age groups.^10^ The literature supports these results, in our study more adenomas, especially conventional tubular adenomas, were observed in the 30-39-year-old age group than in younger age groups (1.91% vs. 0.23%). Anderson et al. reported that individuals younger than 50 years of age have a lower probability of detecting metachronous advanced adenoma in surveillance colonoscopies (3.7% vs 7.3%) compared to patients over 60 years of age, and this rate is very low (0.8%) under the age of 40.^3^ Butterly et al. reported the diminutive polyp rate at 67.5% (1305/1933), the small polyp rate at 25.19% (487/1933), and the polyp rate with advanced histology for polyps smaller than 10 mm at 40.9% (45/110).^11^

In this study, the number of tubular adenomas in the 50-59 age group was three times higher than in the 40-49 age group. We found an increase with age in conventional adenomas and sessile serrated lesions. Cha et al., in a study that included 141 patients younger than 40 years of age, found that the mean age was 34, and 48% of all patients were male. These patients were included in the study on the condition that there was at least one polyp, and they claimed that genetic factors may lie behind the polyp detection in such a young patient. While the uncertainty regarding the application of surveillance colonoscopic screening applied to elderly patients to younger patients continues, it has been suggested that surveillance colonoscopy for young people should be discussed, considering that the development of advanced neoplasia within five years after negative colonoscopy in elderly individuals is 1.4%.^8^ Gupta et al., 68% of all polyps were diminutive polyps, 19% were small polyps and 12% were large polyps. They found advanced histology in approximately 0.55% of diminutive polyps and 1.5% of small polyps.^12^ However, they reported no adenocarcinoma in these two groups. In our study, these rates were 1.16% versus 0.78%, and we detected adenocarcinoma in one a small polyp (Table-III). Advanced histology was detected in 26.6% of adenomas, cancer was detected in 0.8%, and the rate of polyps smaller than 10 mm in advanced adenomas was 69.3%.^13^ In another study, the occurrence of advanced histology in diminutive polyps was 4.7% and the rate of high-grade dysplasia and/or intramucosal carcinoma was 1.2%.^14^ Overall, this study enhances our understanding of colorectal adenomas by providing detailed insights into their characteristics, correlations, risk stratification, and age-related patterns, thereby informing clinical practice and guiding future research efforts in this field.

**Table-III:** Distribution of histopathological colorectal epithelial polyps by size.

	≤5 mm (n=804)	6-9 mm (n=265)	10-19 mm (n=158)	≥20 mm (n=42)	Total polyps (n=1269)
Hyperplastic polyp	174 (21.64%)	32 (12.07%)	11(6.96%)	0(0.00)	217 (17.01%)
SSL-D	7(0.87%)	6(2.26%)	10(6.32%)	3(7.42%)	26(2.04%)
TSA	1(0.12%)	1(0.37%)	6(3.79%)	0(0.00)	8(0.63%)
USA	1(0.12%)	0(0.00)	0(0.00)	0(0.00)	1(0.00)
Tubular adenoma	565(70.26%)	197(74.33%)	85(53.79%)	19(45.23%)	839(68.23%)
Tubulovillous adenoma	17(2.11%)	15(5.66%)	22(13.92%)	10(23.80%)	64(5.04%)
Villous adenoma	3(0.37%)	0(0.00)	2(1.26%)	2(4.76%)	7(0.55%)
Advanced adenoma (and intramucosal ca)	6(0.74%)	2(0.75%)	8(5.06%)	6(14.28%)	22(1.73)
Invasive adenocarcinoma	0(0.00)	1(0.37%)	1(0,63%)	1(2.38%)	3(0.23%)
Total No, epithelial polyps cases	493	107	80	20	700

### Limitations of the study

First, our study was not intended to cover the entire general population, but only included patients who visited a tertiary care hospital during randomized screenings and without ethnic discrimination. For this reason, care should be taken by considering this situation while making general judgments about the research. Another limitation was the periodical difference between the colonoscopy times of the patients since it was a retrospective study. The third limitation was that each endoscopist did not know the polyp or adenoma detection rate, sensitivity, and withdrawal times.

## CONCLUSION

Although there are limitations, the causes of which are detailed below, the incidence of adenomas was higher in people over 50 years old, although young adenomas began to increase from the age of 40-49. Eighty-one percent of all epithelial polyps were less than 10 mm, and the incidence of advanced adenomas was 1.73%. The prevalence of detection of intramucosal carcinoma in diminutive polyps (≤5 mm) was remarkably high (0.77%). Suggestions should be made for endoscopists to try to remove all polyps when they meet and to be aware of the presence of synchronized and metachronized polyps during baseline colonoscopy. The results of this study are valuable for both clinical implications and future public health protection as they have important implications for follow-up and management.
